# Supplementary material for: Subtleties in Clathrin heavy chain binding boxes provide selectivity among adaptor proteins of budding yeast
Source: Nat Commun. 2024 Nov 7;15:9655. doi: 10.1038/s41467-024-54037-z (PMC11543927; doi:10.1038/s41467-024-54037-z)
Supplement: Supplementary file 1 — Supplementary Information [file 41467_2024_54037_MOESM1_ESM.pdf]

Supplementary information for “Subtleties in Clathrin Heavy Chain Binding Boxes provide selectivity among Adaptor Proteins of Budding Yeast”

Lucas A. Defelipe[1,2], Katharina Veith[1,2], Osvaldo Burastero[1,2] ,Tatiana Kupriianova[1,2], Isabel Bento[1], Michal Skruzny[3,4§], Knut Kölbel[2,5,6], Charlotte Uetrecht[2,5,6,7], Roland Thuenauer[2,5,8] and Maria M. García-Alai[1,2*]

1. European Molecular Biology Laboratory - Hamburg Unit, Notkestraße 85, 22607 Hamburg, Germany.
2. Centre for Structural Systems Biology, Notkestraße 85, 22607 Hamburg, Germany.
3. Cell Biology and Biophysics Unit, European Molecular Biology Laboratory, Heidelberg, Germany.
4. Department of Systems and Synthetic Microbiology, Max Planck Institute for Terrestrial Microbiology, 35043 Marburg, Germany.
5. Leibniz Institute of Virology (LIV), Martinistraße 52, 20251 Hamburg, Germany.
6. Deutsches Elektronen Synchrotron - DESY, Notkestraße 85, 22607 Hamburg, Germany.
7. Institute of Chemistry and Metabolomics, University of Lübeck, Ratzeburger Allee 160, 23562 Lübeck, Germany.
8. Technology Platform Light Microscopy (TPLM), Universität Hamburg (UHH), Notkestraße 85, 22607 Hamburg, Germany

* To which correspondence should be addressed: maria.garcia@embl-hamburg.de

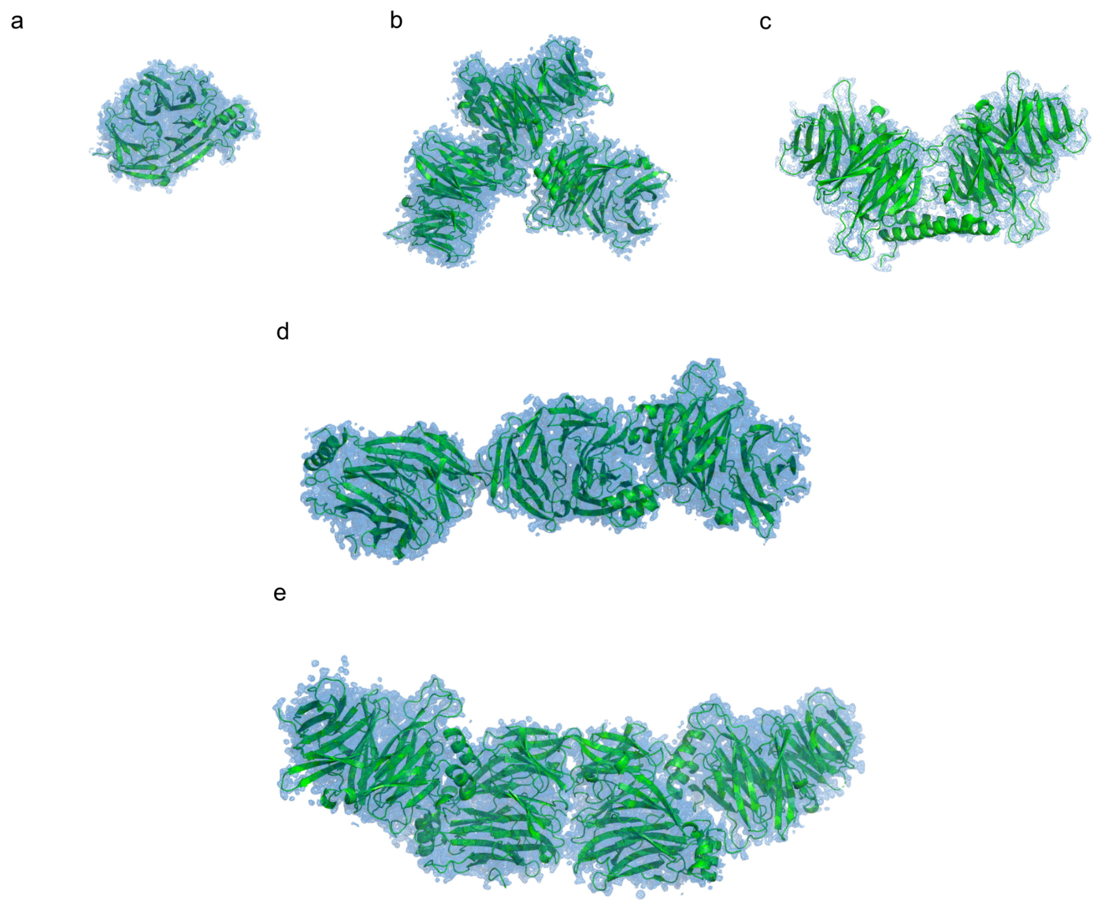

Supplementary Figure 1. Density map ($2F_o$-F_c) of CHC in complex with different peptides. a) Ent1, b) Ent 2, c) APL2, d) Ent5 and e) YAP1801. Density is shown as a blue mesh at a contour level of 1σ (standard deviation) above the mean electron density.

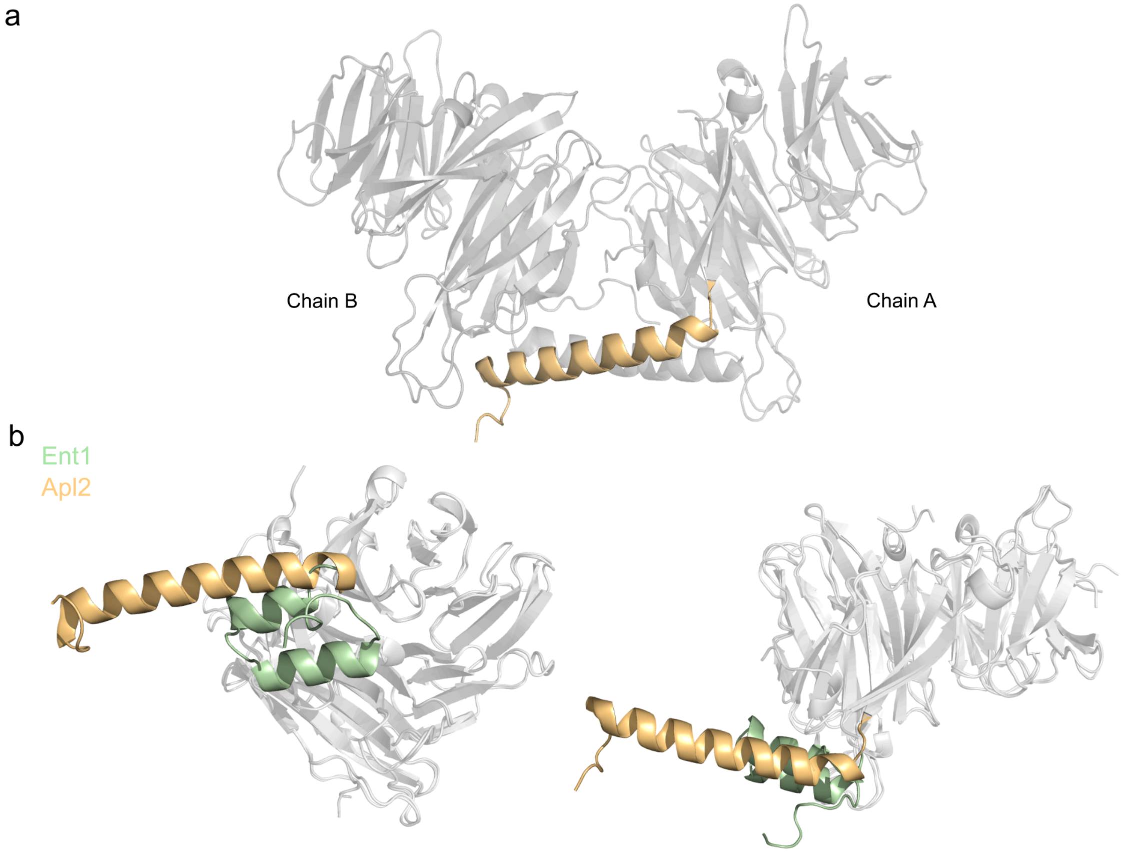

Supplementary Figure 2. a) Crystallographic dimer of NTD complexed with the Apl2.1 peptide showing an alternative conformation of the C-terminal helix. b) Comparison of the NTD C-terminal helix for the Ent1 (green) and Apl2 (orange) bound structures.

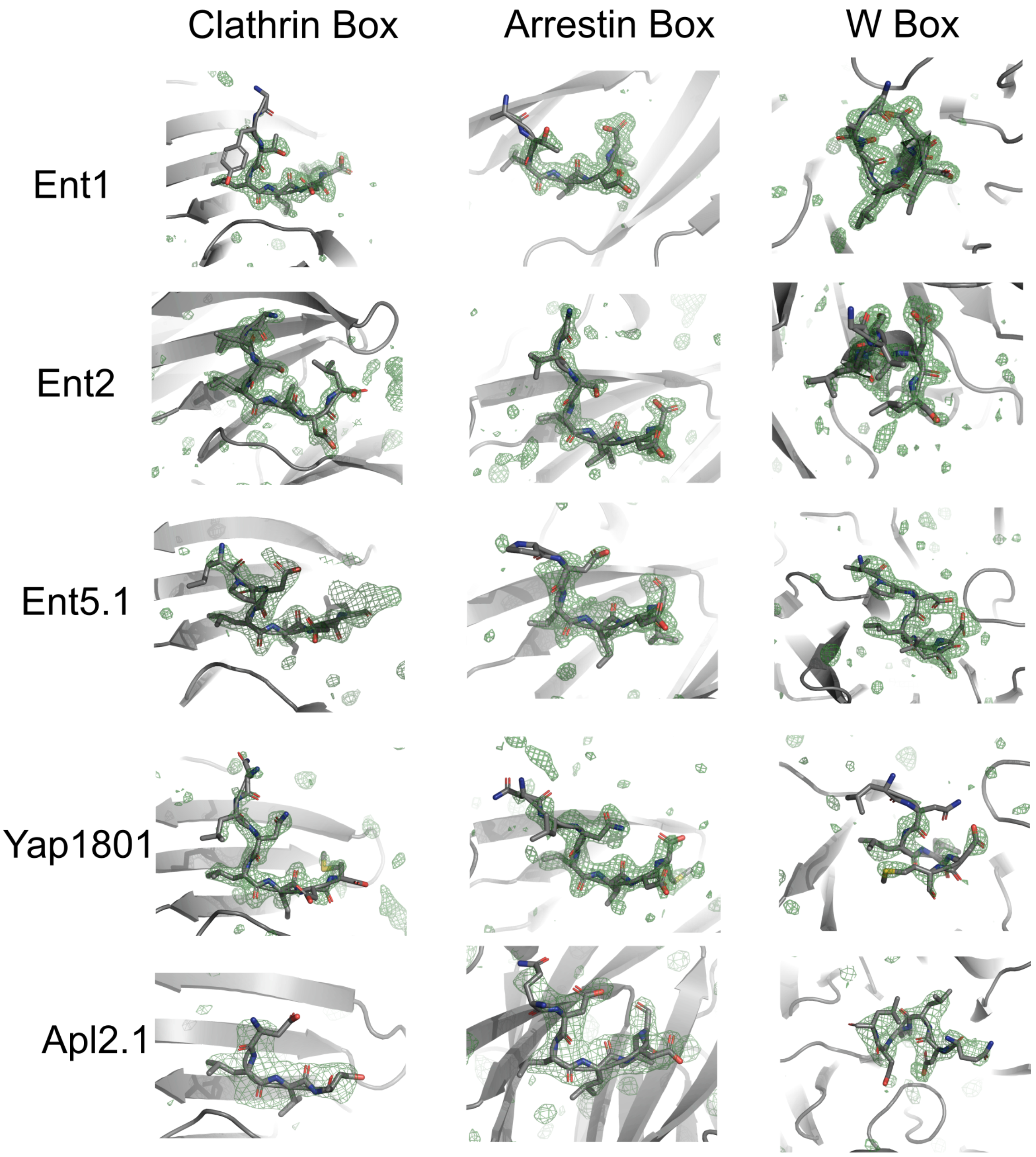

Supplementary Figure 3. Omit map of each ScCHC-NTD/Peptide complex. The electron density map (F_o-F_c) surrounding these peptides is shown as a green mesh at a contour level of 3σ (standard deviation) above the mean electron density.

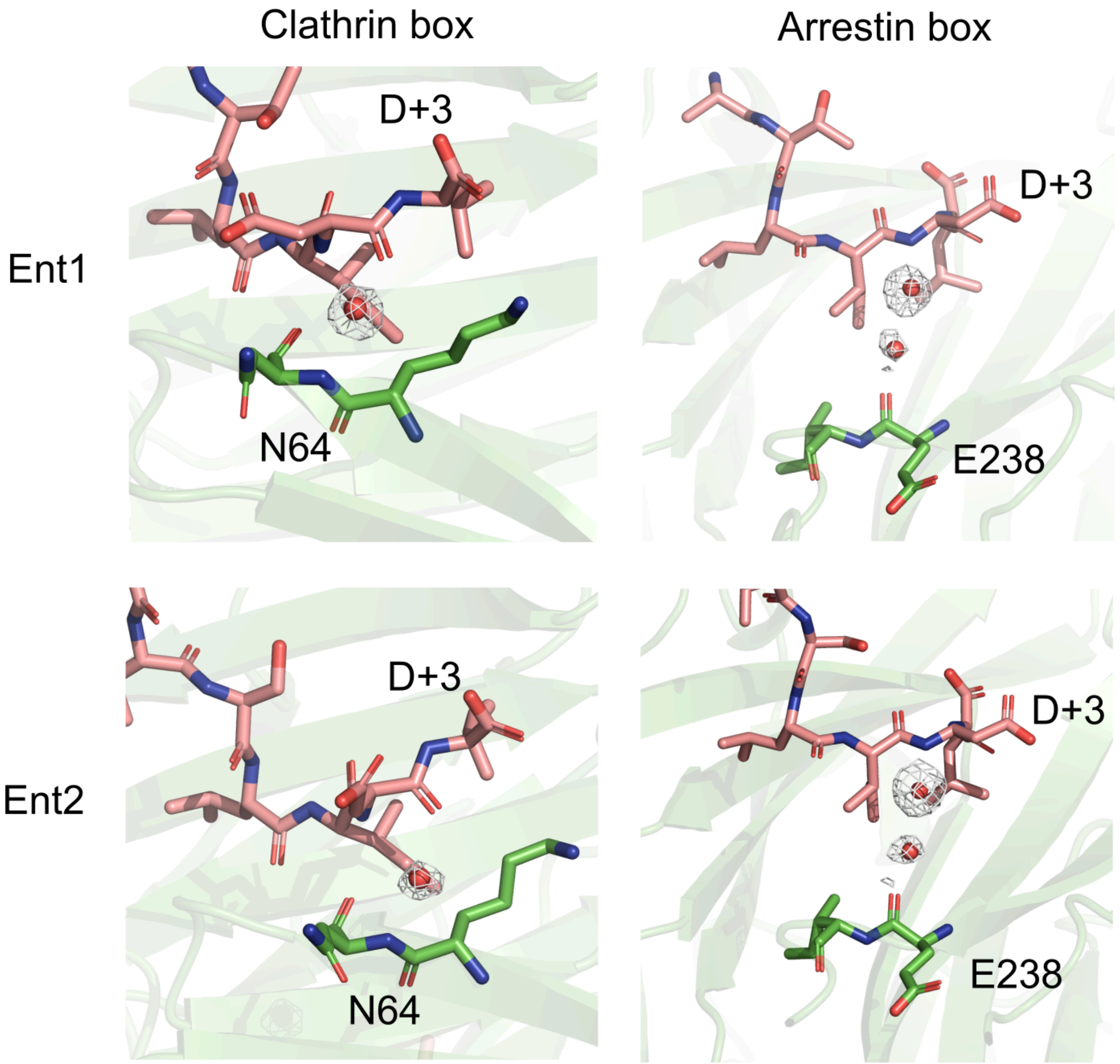

Supplementary Figure 4. Visualization of structural waters within NTD and Ent1 or Ent2 peptides. The electron density map ($2F_o$-F_c) surrounding these water molecules is depicted as a light blue mesh at a contour level of 1σ (standard deviation) above the mean electron density.

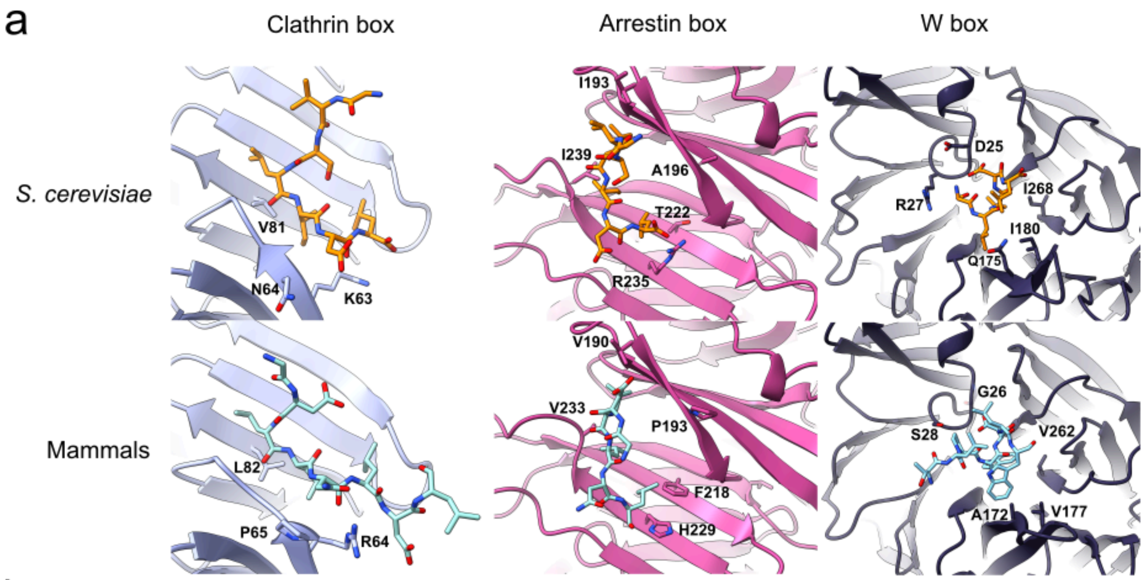

b

```
ScChc NTD    1 -MSDLPIEFTELVDLMSLGISPQFLDFRSTTFESDHFVTVRETKDGTNSVAIVDLAKGNEVT  61
HsChc NTD    1 MAQILPIRFQEHLQLQNLGINPANIGFSTLTMESDKFICIREKVGEQAQVVIIDMNDPSNPI  62

ScChc NTD   62 RKNMGGDSAIMHPSQMVISVRANGTIVQIFNLETKSKLKSFTLDEPVIFWRWLSETTLGFVT 123
HsChc NTD   63 RRPISADSAIMNPASKVIALKA-GKTLQIFNIEMKSKMKAHTMTDDVTFWKWISLNTVALVT 123

ScChc NTD  124 ARSILTSNVFDGNVNAKPQLLTLRHANLNNTQIINFVANKNLDWFAVVGILQENGRIAGRIQ 185
HsChc NTD  124 DNAVYHWS---MEGESQPVKMFDRHSSLAGCQIINYRTDAKQKWLLLTGISAQQNRVVGAMQ 182

ScChc NTD  186 LFSKQRNISQAIDGHVAIFTNILLEGNGSTPVQVFVTGNRNATTGAGELRIIEIDHDASLPS 247
HsChc NTD  183 LYSVDRKVSQPIEGHAASFAQFKMEGNAEESTLFCF-AV--RGQAGGKLHIIEVGTPPTGNQ 241

ScChc NTD  248 QYQKETTDIFFPPDATNDFPIAVQVSEKYGIIYLLTKYGFIHLYELETGTNLFVNRITAESV 309
HsChc NTD  242 PFPKKAVDVFFPPEAQNDFPVAMQISEKHDVVFLITKYGYIHLYDLETGTCIYMNRISGETI 303

ScChc NTD  310 FTAAPYNHENGIACINKKGQVLAVEISTSQIVPYILNKLSNVALALIVATRGGLPGADDL   369
HsChc NTD  304 FVTAPHEATAGIIGVNRKGQVLSVCVEEENIIPYITNVLQNPDLALRMAVRNNLAGAEEL   363
```

Supplementary Figure 5. Structural comparison of *S. cerevisiae* and mammalian Chc boxes. a) Crystal structures of *S.cerevisiae* and mammalian (*Bos taurus* and human) NTD: Clathrin box (violet), Arrestin box (pink) and W-box (gray). *S. cerevisiae*: Cartoon representation of the three boxes bound to the Ent2 peptide. Mammalian: *Bos taurus* Clathrin and Arrestin boxes bound to human AP-2 Beta peptide (PDB 5M5R) and human W-box bound to human Amphiphisin peptide (PDB 1UTC). b) Sequence alignment between *S. cerevisiae* and *Bos taurus* NTD.

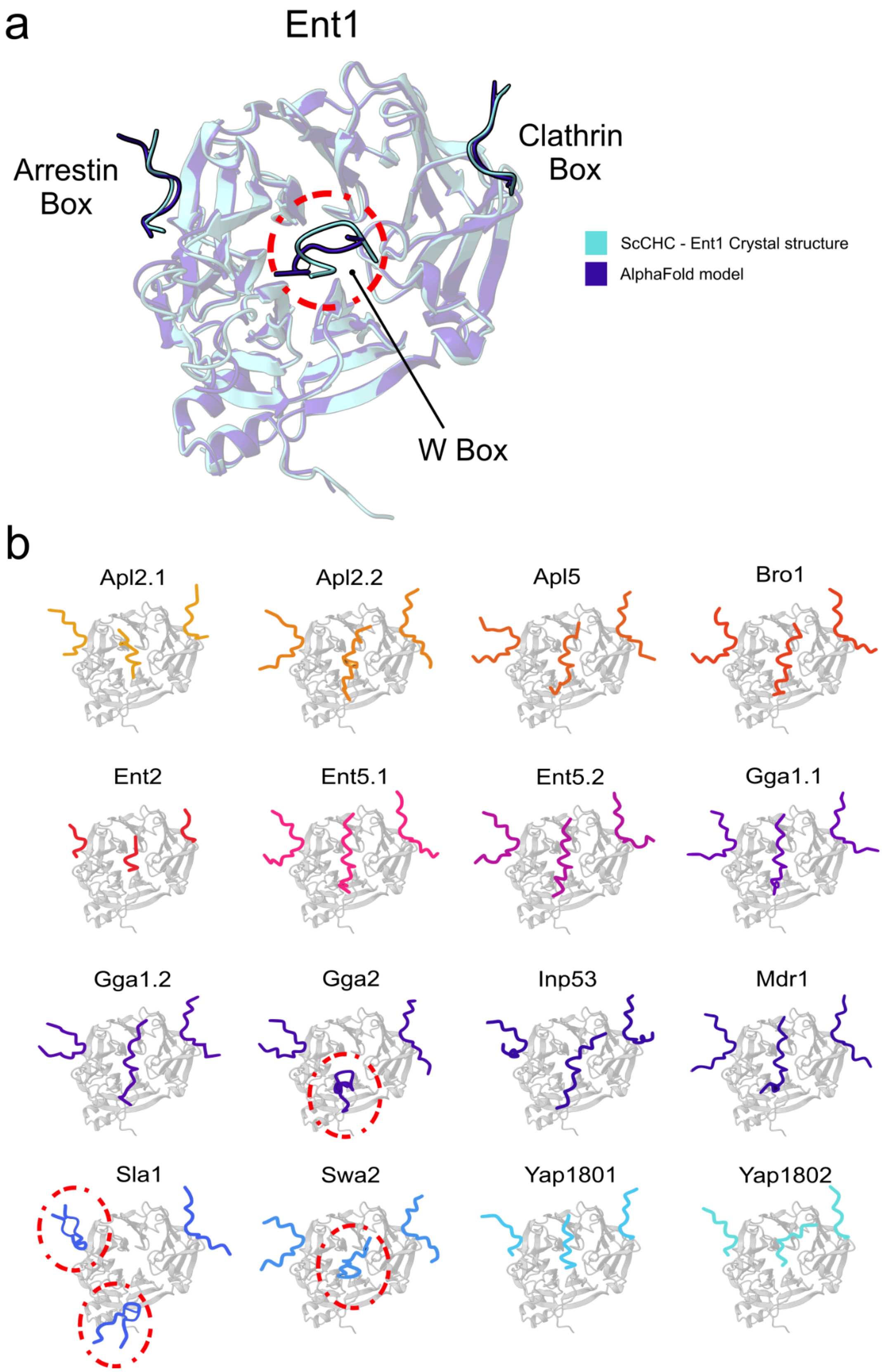

Supplementary Figure 6. AlphaFold2 predictions for the NTD CBM complexes. a) Superposition of the crystal structure of NTD bound to Ent1 peptide (cyan) and the AF2 best model (blue). b) AF2 best predictions for NTD in complex with various CBM-containing peptides. Dashed red circles highlight wrong predictions in all five models.

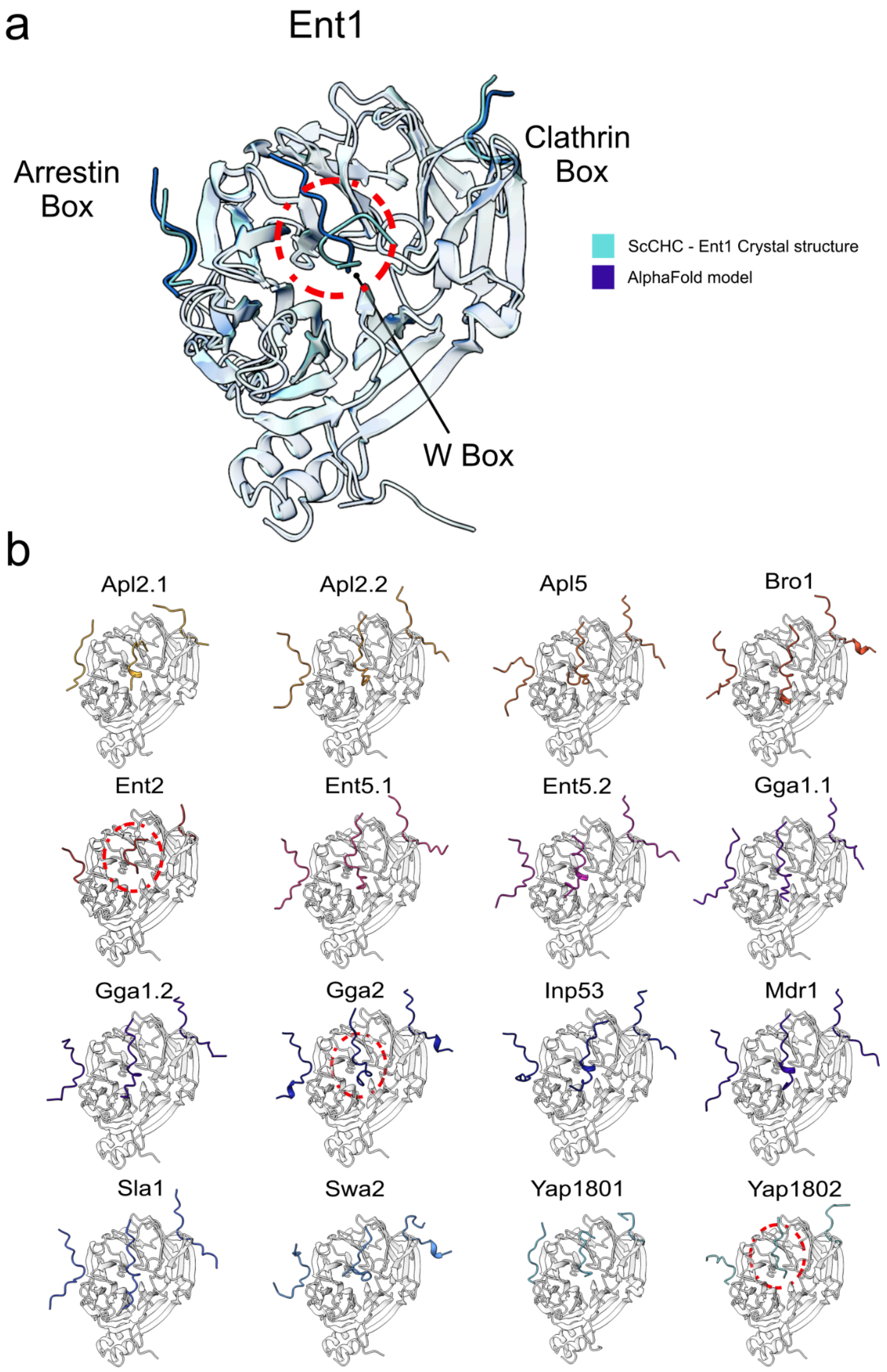

Supplementary Figure 7. AlphaFold3 predictions for the NTD CBM complexes. a) Superposition of the crystal structure of NTD bound to Ent1 peptide (cyan) and the AF3 best model (blue). b) AF3 best predictions for NTD in complex with various CBM-containing peptides. Dashed red circles highlight wrong predictions in all five models.

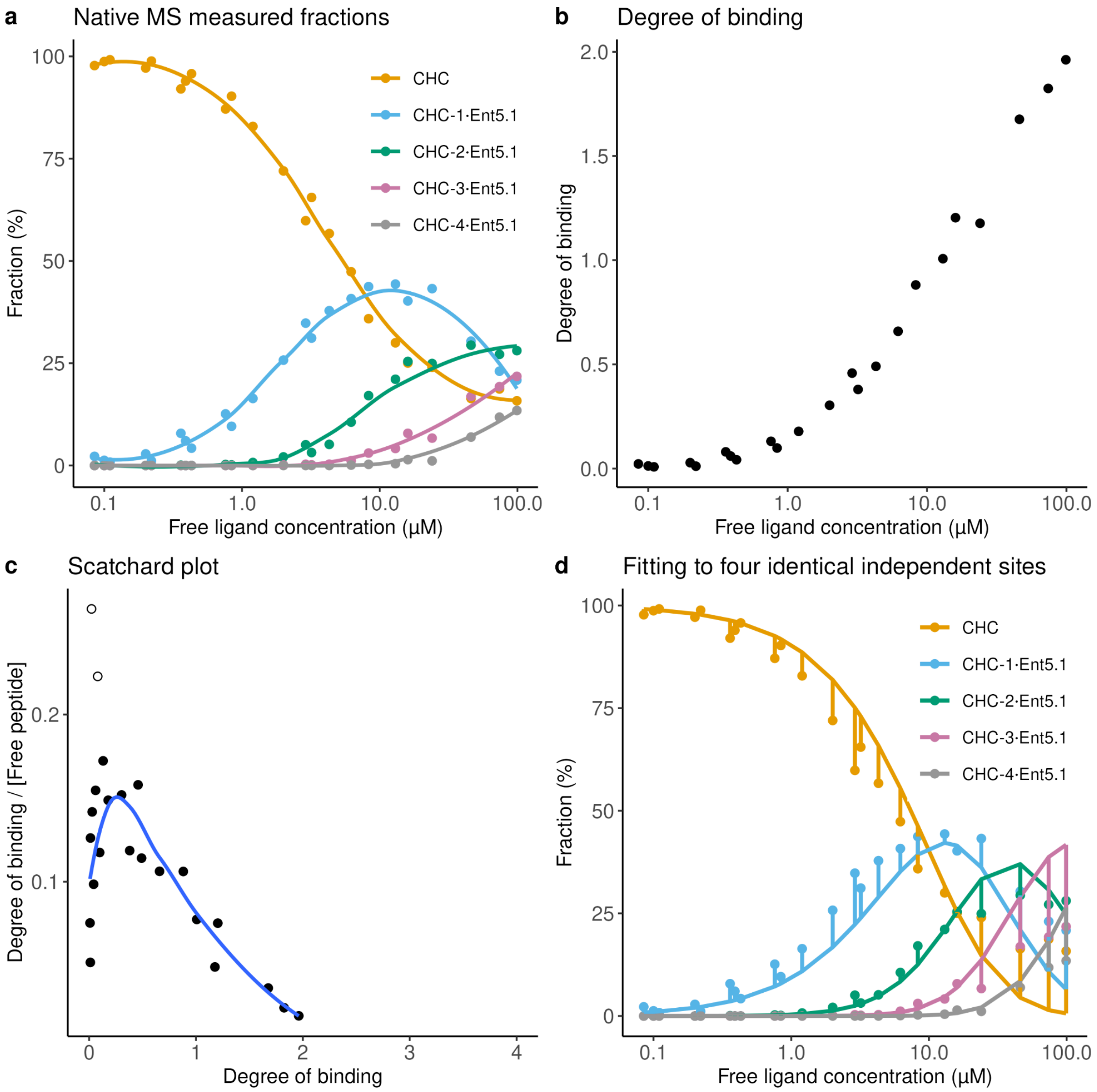

Supplementary Figure 8. Native mass spectrometry (native MS) analysis of the interaction between Clathrin heavy chain (CHC) and the Ent5.1 peptide. See Section Supplementary Methods. a) Points: measured fraction versus free ligand concentration (μM). Lines: Smoothed lines based on the locally estimated scatterplot smoothing method (LOESS). b) Degree of binding versus free peptide (ligand) concentration (μM). c) Scatchard plot (free peptide concentration in μM). Blue line: LOESS-based smoothing without the two empty dots. d) Points: Measured fraction versus free ligand concentration (μM). Lines: Estimation based on a microscopic K_D of 39.1 μM assuming four independent sites with the same binding affinity. Vertical lines: Difference between the data and the predicted fractions. The microscopic K_D was obtained by performing a global non-linear square fitting of the five different measured fractions.

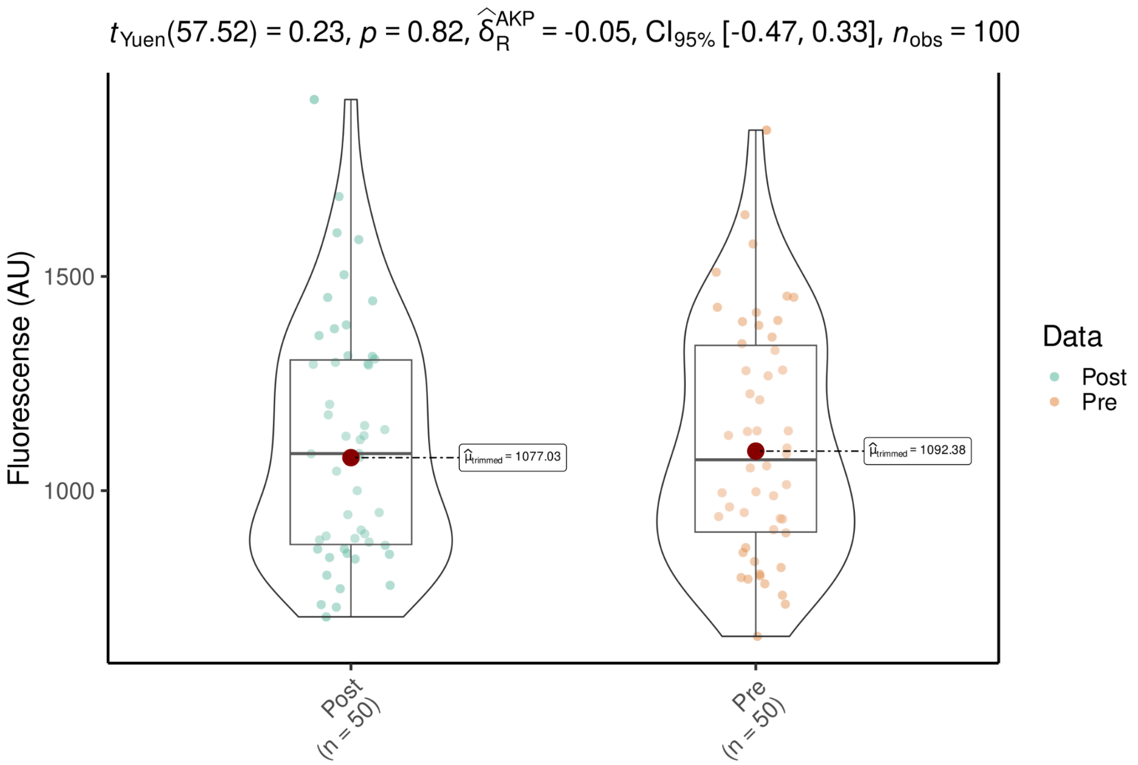

Supplementary Figure 9. FRET Specificity. Fluorescence intensity of Ent1-mNeonGreen-tagged cells excited with the 488 nm laser was measured before (Pre) and after photobleaching (Post) in the mScarlet channel (561 nm and 640 nm lasers). Data were analyzed using Yuen's trimmed means test[1], and no significant differences were observed, demonstrating that the photobleaching process does not affect donor fluorescence.

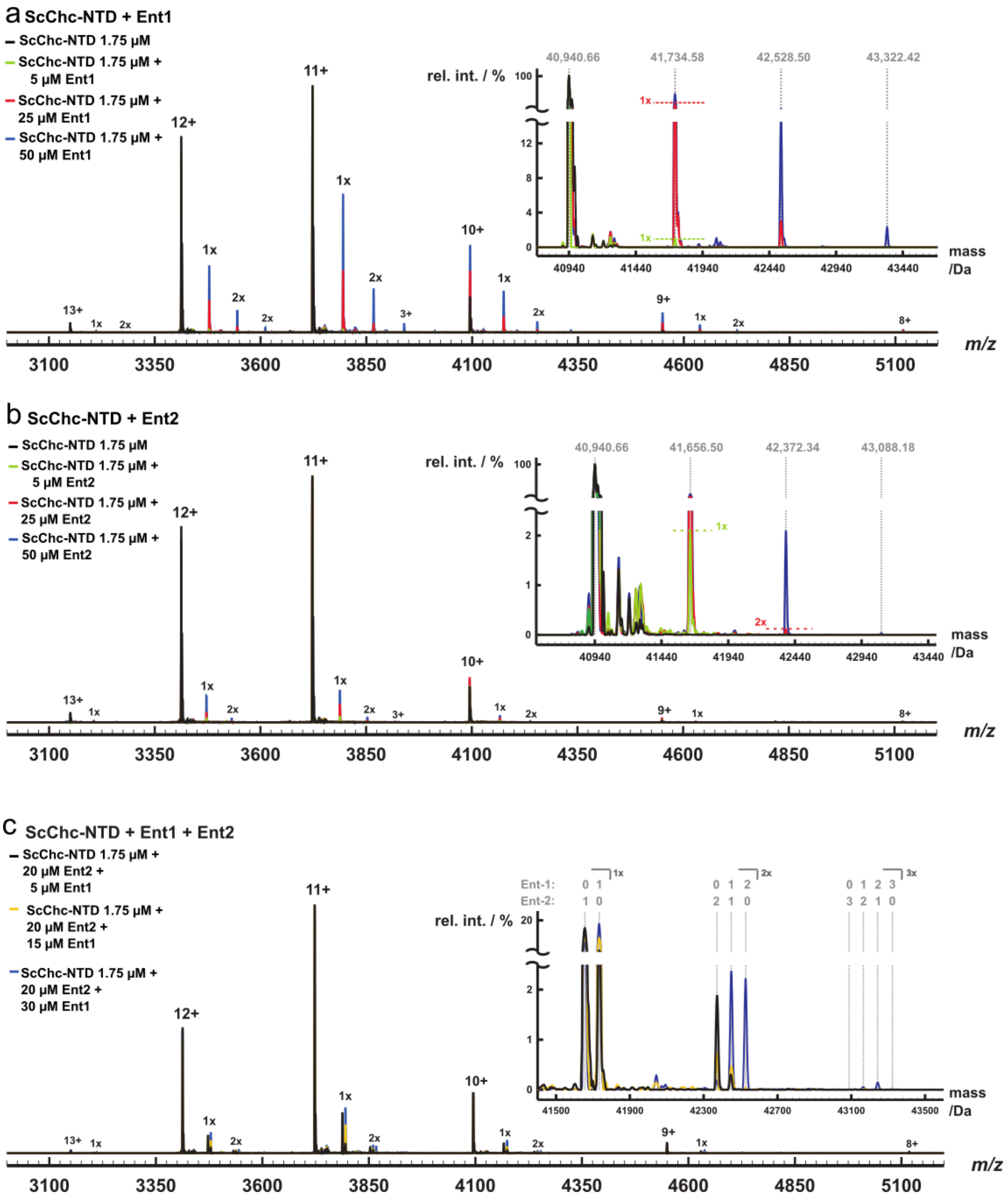

Supplementary Figure 10. Native mass spectrometry spectra. a) Native MS of free ScCHC-NTD (black) and in presence of Ent1 at 5 µM (green), 25 µM (red) and 50 µM (blue). Right, inset of the 11+ ions showing single, double and triple bound ScCHC-NTD with Ent1. b) Native MS of free ScCHC-NTD (black) and in presence of Ent2 at 5 µM (green), 25 µM (red) and 50 µM (blue). Right, inset of the 11+ ions showing single, double and triple bound ScCHC-NTD with Ent2. c) Native MS of ScCHC-NTD in presence of both Ent1 and Ent2. Right, inset of the 11+ ions showing single, double and triple bound ScCHC-NTD with Ent1 and Ent2.

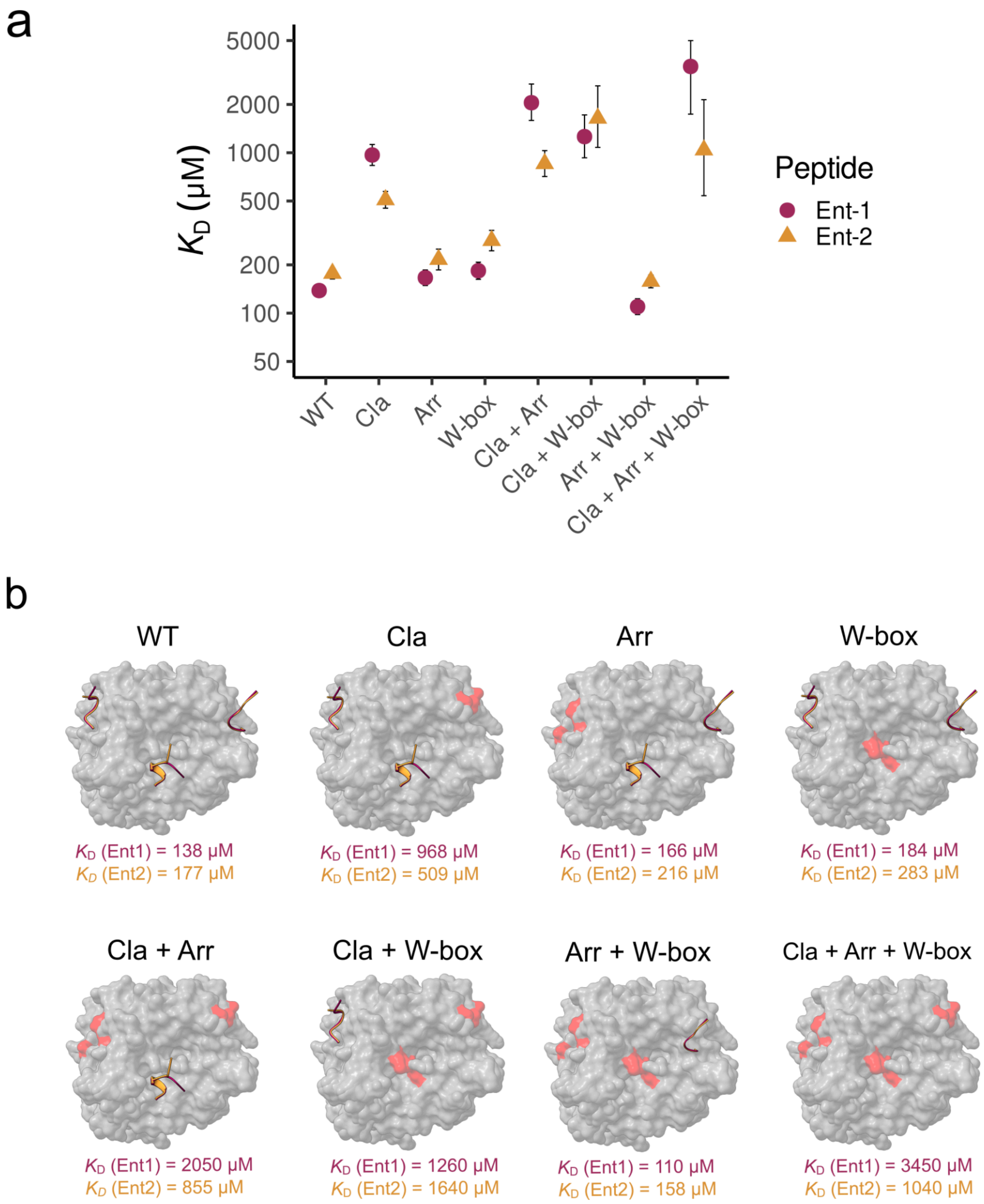

Supplementary Figure 11. Binding Affinities of Ent1 and Ent2 for WT Chc-NTD and Mutants. a) Determination of apparent dissociation constants (K_D^{App}) by nano Differential Scanning Fluorimetry (nDSF) based on T_m shifts for Ent1 and Ent2 peptides with WT NTD and mutants at Clathrin (K63E, I87D, Q89A and K98E), Arrestin (Q195A, I197T and K251E), and W-box (F26A and Q155A) binding sites. Data is shown as mean asymmetric IC95 as implemented in FoldAffinity (spc.embl-hamburg.de). b) Schematic representation of these K_D^{App} values mapped onto the NTD structure (surface in gray). The presence of a peptide (Ent1 in red and Ent2 in yellow) indicates assumed peptide binding. Mutated residues are shown in red.

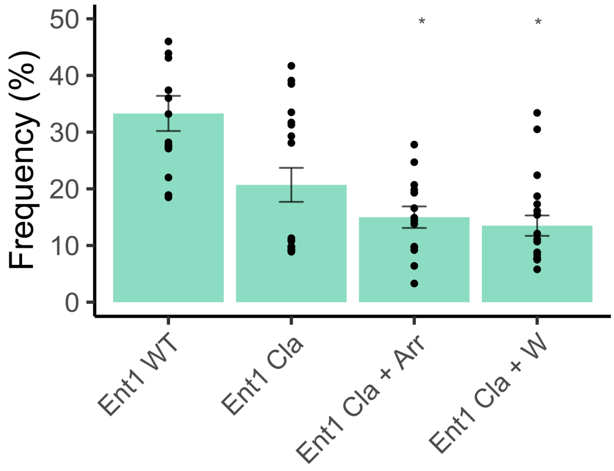

Supplementary Figure 12. Abp presence in Ent1-mNeonGreen endocytic sites of different CHC mutants. * indicates a p-value of 8.1e^{-5} , denoting statistical significance in a two-tailed Mann–Whitney U-test corrected for multiple comparisons (WT n=15, Cla n=18, Cla + Arr n=14, Cla + W n=19).

Supplementary Table 1. Proteins from *Saccharomyces cerevisiae* exhibiting the Clathrin Binding Motifs (CBMs). This table enumerates proteins identified in *Saccharomyces cerevisiae* that contain the designated motif, with uppercase letters delineating the motif itself and lowercase letters representing the sequences flanking the motif within the protein sequence.

UniProt ID	Protein Name	Gene Name	Sequence	Seq Start	Seq Stop	Type of motif
P53309	Clathrin coat assembly protein AP180B	YAP1802	enpNLIDI	564	568	Canonical
P38856	Clathrin coat assembly protein AP180A	YAP1801	nnINLIDM	633	637	Canonical
Q08562	ATP-dependent helicase ULS1	ULS1	eviSLLDLpni	411	415	Canonical
Q01476	Ubiquitin carboxyl-terminal hydrolase 2	UBP2	kdtSLIDLeme	876	880	Canonical
Q06677	Auxilin-like clathrin uncoating factor SWA2	SWA2	iedSLLDFseg	333	337	Canonical
Q08204	Structural maintenance of chromosomes protein 5	SMC5	mtSLIDLgry	3	7	Canonical
P32790	Actin cytoskeleton-regulatory complex protein SLA1	SLA1	smqDLLDLqpl	802	806	Canonical

P38814	Protein SBE22	SBE22	snaSLLDFnem	406	410	Canonical
P43565	Serine/threonine-protein kinase RIM15	RIM15	lsfSLLDIsrs	1066	1070	Canonical
P53258	GTPase-activating protein GYP2	MDR1	eevNLIDLsdd	759	763	Canonical
Q08001	Membrane-anchored lipid-binding protein LAM6	LAM6	ahaTLLDIpak	674	678	Canonical
Q04746	Nuclear fusion protein KAR5	KAR5	ktgSLIDF	500	504	Canonical
Q12271	Polyphosphatidylinositol phosphatase INP53	INP53	sesSLLDIdpi	914	918	Canonical
P39996	Glutathione transferase 3	GTT3	tnfNLLDFstd	140	144	Canonical
P38817	ADP-ribosylation factor-binding protein GGA2	GGA2	neiNLIDFndl	351	355	Canonical
Q06336	ADP-ribosylation factor-binding protein GGA1	GGA1	kelNLIDFddd	336	340	Canonical
Q06336	ADP-ribosylation factor-binding protein GGA1	GGA1	sgiDLLDFdsq	400	404	Canonical
Q99369	Family of serine hydrolases 3	FSH3	ledDLLDMids	255	259	Canonical
Q03769	Epsin-5	ENT5	sipDLIDLdds	292	296	Canonical
Q03769	Epsin-5	ENT5	kidDLLDWdgp	354	358	Canonical
Q05785	Epsin-2	ENT2	qgvSLIDL	609	613	Canonical
Q12518	Epsin-1	ENT1	rgyTLIDL	450	454	Canonical
P48582	Vacuolar-sorting protein BRO1	BRO1	pqpSLLDIddt	537	541	Canonical
P53958	Protein BOP3	BOP3	kskSLLDIifg	26	30	Canonical
P53858	Protein BNI4	BNI4	tspNLIDIdgs	161	165	Canonical
Q08951	AP-3 complex subunit delta	APL5	nskDLLDLnee	745	749	Canonical

P36000	AP-1 complex subunit beta-1	APL2	vsqDLLDLf	721	725	Canonical
P36000	AP-1 complex subunit beta-1	APL2	nddVLLDFder	637	641	Canonical
P38817	ADP-ribosylation factor-binding protein GGA2	GGA2	vndLLGDLtdl	385	389	Arrestin
P34758	Protein SCD5	SCD5	ssdILGNLqsl	850	854	Arrestin

Supplementary Table 2. Percentage of identity in the N-terminal domain region of the clathrin heavy chain (isoform 1) among selected mammalian sequences compared to the human sequence. The percentages were retrieved by running Blastp (https://blast.ncbi.nlm.nih.gov/Blast.cgi?PAGE=Proteins) (accessed on 07/03/2024) with the following non-default parameters: 'Database' - 'refseq_protein', 'Organism' - 'Mammalia' and 'Matrix' - 'BLOSUM45'. The query sequence was the first 369 residues of the Clathrin heavy chain (UniprotKb ID Q00610). The results were filtered to have a coverage greater than 99 %.

**Description**	**Scientific Name**	**Per. ident**
PREDICTED: clathrin heavy chain 1 isoform X1 [Bison bison bison]	Bison bison bison	100
clathrin heavy chain 1 isoform X2 [Rattus norvegicus]	Rattus norvegicus	100
clathrin heavy chain 1 isoform X3 [Sus scrofa]	Sus scrofa	100
clathrin heavy chain 1 isoform X3 [Rattus norvegicus]	Rattus norvegicus	100
clathrin heavy chain 1 isoform X3 [Mirounga angustirostris]	Mirounga angustirostris	100
clathrin heavy chain 1 isoform X4 [Odocoileus virginianus texanus]	Odocoileus virginianus texanus	100
clathrin heavy chain 1 isoform X3 [Pongo abelii]	Pongo abelii	100

PREDICTED: clathrin heavy chain 1 isoform X3 [Capra hircus]	Capra hircus	100
clathrin heavy chain 1 isoform X4 [Pongo abelii]	Pongo abelii	100
clathrin heavy chain 1 isoform X3 [Bubalus kerabau]	Bubalus kerabau	100
clathrin heavy chain 1 isoform X3 [Castor canadensis]	Castor canadensis	100
clathrin heavy chain 1 isoform X3 [Microcebus murinus]	Microcebus murinus	100
clathrin heavy chain 1 isoform X1 [Leptonychotes weddellii]	Leptonychotes weddellii	100
clathrin heavy chain 1 isoform X3 [Odocoileus virginianus texanus]	Odocoileus virginianus texanus	100
clathrin heavy chain 1 isoform X2 [Delphinapterus leucas]	Delphinapterus leucas	100
clathrin heavy chain 1 isoform X2 [Myotis lucifugus]	Myotis lucifugus	100
clathrin heavy chain 1 isoform X3 [Lagenorhynchus obliquidens]	Lagenorhynchus obliquidens	100
clathrin heavy chain 1 isoform X3 [Pteronotus parnellii mesoamericanus]	Pteronotus parnellii mesoamericanus	100
clathrin heavy chain 1 isoform X1 [Cynocephalus volans]	Cynocephalus volans	100
clathrin heavy chain 1 isoform X1 [Erinaceus europaeus]	Erinaceus europaeus	100
clathrin heavy chain 1 isoform X2 [Carlito syrichta]	Carlito syrichta	100
clathrin heavy chain 1 [Bos taurus]	Bos taurus	100
PREDICTED: clathrin heavy chain 1 [Bos indicus]	Bos indicus	100
clathrin heavy chain 1 isoform 1 [Mus musculus]	Mus musculus	100

Supplementary Table 3. Crystallographic data collection and refinement. Values for the highest resolution shell are shown in parentheses.

NTD Complex	Ent1	Ent2	Ent5	YAP1801	APL2
PDB ID	9EYT	9EXG	9EX5	9EXF	9EXT
**Data collection**					
Beamline	PETRAIII/P13	PETRAIII/P14	PETRAIII/P14	PETRAIII/P14	PETRAIII/P14
Space Group	P 43 21 2	P 21 21 21	C 2 2 21	P 1 21 1	P 41 21 2
Cell Dimensions					
a, b, c (Å)	56.31, 56.31, 254.60	46.50, 94.29, 269.03	77.00, 133.45, 285.53	51.08, 89.74, 188.12	141.78, 141.78, 168.597
α, β, γ (°)	90.0, 90.0, 90.0	90.0, 90.0, 90.0	90.0, 90.0, 90.0	90.0, 90.39, 90.0	90.0, 90.0, 90.0
Resolution (Å)	63.73 – 1.74 (1.77-1.74)	67.35 – 1.74 (1.77-1.74)	71.38 – 2.01 (2.05-2.01)	65.01 – 1.95 (1.98-1.95)	100.3 - 2.75 (2.85-2.75)
Rpim	0.019 (0.545)	0.037 (0.457)	0.049 (0.517)	0.063 (0.751)	0.022 (0.785)
<I/σI>	27.2 (1.78)	16.7 (1.80)	15.1 (1.5)	11.0 (1.79)	30.0 (1.6)
CC 1/2	1 (0.843)	0.999 (0.788)	0.999 (0.533)	0.998 (0.553)	1 (0.589)
Completeness (%)	100 (99.9)	99.8 (99.8)	95.9 (34.9)	94.4 (86.3)	99.99 (100)
Redundancy	26 (27.5)	13.1 (12.9)	12.9(3.5)	7.7 (7.1)	13.1 (2.8)
**Refinement**					
No. of reflections (work/free)	43582/2114	121660/6253	93850/4607	116855/5916	45265/2259
Rwork/Rfree	0.185/0.230	0.191/0.225	0.182/0.214	0.183/0.214	0.228/0.258
Ramachandran favoured regions (%)	96.6	98.1	98.3	98.7	96.51
Ramachandran allowed regions (%)	3.4	1.9	1.7	1.2	3.49
Ramachandran outliers (%)	0	0	0	0.07	0.0
Rotamer outliers (%)	0.91	1.31	1.11	2.22	1.07
Clashscore	1.17	2.00	1.01	2.91	8.62
No. of atoms					
Protein	2844	8511	8452	11485	5698
Petide	151	428	449	281	211
Ligands	0	5	0	1	0
Water	324	929	604	614	6
B-factors (Average)					
Protein	39.59	32.98	42.52	36.85	97.81
Peptide	48.48	37.71	51.28	50.99	114.20
Ligands	-	35.40	-	65.80	-
Water	43.43	35.53	45.74	36.51	92.60
RMS deviations					
Bond lengths (Å)	0.01	0.011	0.01	0.018	0.006
Bond angles (°)	1.7	1.57	1.45	1.83	0.93

Supplementary Table 4. Primers used for QuikChange mutagenesis in both pETM-30-GST-ScCHC-NTD and pRS315-5'UTR CHC-mScarlet plasmids.

Name	Box	Mutation	Type	Sequence
Q89A_Fw	Clathrin	Q89A	Fw	GTTAGAGCAAATGGTACTATCGTGgccATATTTAATTTGGAAACTAAGAGCAAG
Q89A_Rev	Clathrin	Q89A	Rev	CTTGCTCTTAGTTTCCAAATTAAATATGGCCACGATAGTACCATTTGCTCTAAC
Q195A_Fw	Arrestin	Q195A	FW	CAATTATTCTCAAAACAACGTAACATCTCCgccGCTATCGACGGTCATGTTGCTATC
Q195A_Rev	Arrestin	Q195A	Rev	GATAGCAACATGACCGTCGATAGCGGCGGAGATGTTACGTTGTTTTGAGAATAATTG
Q155A_Fw	W-Box	Q155A	FW	CCTTGAGACACGCTAACTTGAACAATACCgccATTATCAATTTTGTGGCTAACAAAAACC
Q155A_Rev	W-Box	Q155A	Rev	GGTTTTTGTTAGCCACAAAATTGATAATGGCGGTATTGTTCAAGTTAGCGTGTCTCAAGG
F8A_Fw	Royle	F8A	FW	GAGTGACCTACCCATTGAAgccACCGAATTGGTCGATCTGATGTCCTTAGG
F8A_Rev	Royle	F8A	Rev	CCTAAGGACATCAGATCGACCAATTCGGTGGCTTCAATGGGTAGGTCACTC
K63E_Fw	Clathrin	K63E	Fw	GGCAATGAAGTGACAAGGGAGAATATGGGCGGTGATTCTGCTATCATG
K63E_Rev	Clathrin	K63E	Rev	CATGATAGCAGAATCACCGCCCATATTCTCCCTTGTCACTTCATTGCC
K98E_Fw	Clathrin	K98E	Fw	CAGATATTTAATTTGGAAACTAAGAGCGAGTTAAAGTCTTTTACTTTAGATG
K98E_Rev	Clathrin	K98E	Rev	CATCTAAAGTAAAAGACTTTAACTCGCTCTTAGTTTCCAAATTAAATATCTG
R191E_Fw	Arrestin	R191E	Fw	GAATTCAATTATTCTCAAAACAAGAGAACATCTCCCAAGCTATCGACGGTC
R191E_Rev	Arrestin	R191E	Rev	GACCGTCGATAGCTTGGGAGATGTTCTCTTGTTTTGAGAATAATTGAATTC
K251E_Fw	Arrestin	K251E	Fw	GCTTCATTGCCTTCTCAATATCAAGAGGAAACTACCGATATTTTCTTTCC
K251E_Rev	Arrestin	K251E	Rev	GGAAAGAAAATATCGGTAGTTTCCTCTTGATATTGAGAAGGCAATGAAGC
R27E_Fw	W-box	R27E	Fw	GGAATTTCCCCTCAATTCCTTGACTTCGAATCAACTACTTTCGAGAGTGAC
R27E_Rev	W-box	R27E	Rev	GTCACTCTCGAAAGTAGTTGATTCGAAGTCAAGGAATTGAGGGGAAATTCC
K326E_Fw	W-box	K326E	Fw	GAACGGTATTGCATGCATCAATGAAAAAGGTCAAGTTTTAGCAGTAGAG
K326E_Rev	W-box	K326E	Rev	CTCTACTGCTAAAACTTGACCTTTTTCATTGATGCATGCAATACCGTTC
R191E+Q195A_Fw	Arrestin	R191E+Q195A	Fw	CAATTATTCTCAAAACAAGAGAACATCTCCGCCGCTATCGACGGTC
R191E+Q195A_Rev	Arrestin	R191E+Q195A	Rev	GACCGTCGATAGCGGCGGAGATGTTCTCTTGTTTTGAGAATAATTG
F26A_Fw	Wbox	F26A	Fw	GGAATTTCCCCTCAATTCCTTGACGCCAGATCAACTACTTTCGAG
F26A_Rev	WBox	F26A	Rev	CTCGAAAGTAGTTGATCTGGCGTCAAGGAATTGAGGGGAAATTCC
I197T_Fw	Arrestin	I197T	Fw	CTCAAAACAACGTAACATCTCCgccGCTaccGACGGTCATGTTG
I197T_Rev	Arrestin	I197T	Rev	CAACATGACCGTCGGTAGCGGCGGAGATGTTACGTTGTTTTGAG
I193A_Fw	Arrestin	I193A	Fw	CTTAGTTTCCAAATTAAATATGGCCACGTCAGTACCATTTGCTCT
I193A_Rev	Arrestin	I193A	Rev	AGAGCAAATGGTACTGACGTGGCCATATTTAATTTGGAAACTAAG
I193A_Fw	Arrestin	I193A	Fw	CTCAAAACAACGTAACgccTCCgccGCTATCGACGGTCATGTTG
I193A_Rev	Arrestin	I193A	Rev	CAACATGACCGTCGATAGCGGCGGAGGCGTTACGTTGTTTTGAG
F91Q_Fw	Clathrin	F91Q	Fw	AGAGCAAATGGTACTATCGTGgccATAcaaAATTTGGAAACTAAG
F91Q_Rev	Clathrin	F91Q	Rev	CTTAGTTTCCAAATTTTGTATGGCCACGATAGTACCATTTGCTCT
I87D_Fw	Clathrin	I87D	Fw	AGAGCAAATGGTACTgacGTGgccATATTTAATTTGGAAACTAAG
I87D_Rev	Clathrin	I87D	Rev	CTTAGTTTCCAAATTAAATATGGCCACGTCAGTACCATTTGCTCT
I87D+Q89A+K98E	Clathrin	I87D+Q89A+K98E	Fw	CAAATGGTACTgacGTGgccATATTTAATTTGGAAACTAAGAGCgagTTAAAG
I87D+Q89A+K98E	Clathrin	I87D+Q89A+K98E	Rev	CTTTAACTCGCTCTTAGTTTCCAAATTAAATATGGCCACGTCAGTACCATTTG

Supplementary Table 5. Primers used for endogenous tagging of ABP1 with mTurquoise2 and to knock out CHC.

Name	Mutation	Type	Sequence
ABP1_S3	ABP1_mTurquoise2	Fw	GAATTTGTCGACGATGACTGGTGGCTAGGGGAACTAGAGAAAGACGGCTCAAAAGGTCTCTTCCCCAGCAATTATGTGTCTTTGGGCAACcgtacgctgcaggtcgac
ABP1_S2	ABP1_mTurquoise3	Rev	CGTGAAAGTATCTATACAAAAGCTTTAACGTCTCTGTAAGTATTTTTTTACGTAAGAATAATATAATAGCATGACGCTGACGTGTGATTatcgatgaattcgagctcg
CHC_S1	CHC_KO	Fw	GAGAAAGAAGATATTCAACTATAAAAATAAATTATCACGAAAGTTCTAACCAATGcgtacgctgcaggtcgac
CHC_S2	CHC_KO	Rev	AAAAAAAAAAATACACGATGGGGTACAGCAAACGAATTATTTTATCCACGTCttaatcgatgaattcgagctcg

Supplementary Table 6. Distribution of Bound Peptides in the Competition Experiment Between Ent1 and Ent2 with Ent1 at a Constant Concentration

[Ent-1] / μM	[Ent-2] / μM	f 0x / %	f 1x / %		f 2x / %			f 3x / %				f 4x / %				
Ent1/Ent2 peptides bound			1/0	0/1	2/0	"1/1"	0/2	3/0	"2/1"	"1/2"	0/3	4/0	"3/1"	"2/2"	"1/3"	0/4
20	0	85.76 ±0.9	12.78 ±0.7	-	1.36 ±0.2	-	-	0.10 ±0.03	-	-	-	-	-	-	-	-
20	5	84.31 ±1.7	11.93 ±1.1	1.82 ±0.2	1.21 ±0.3	0.54 ±0.1	0.03 ±0.01	0.10 ±0.04	0.04 ±0.02	0.003 ±0.003	0.006 ±0.006	-	-	-	-	-
20	10	79.40 ±1.0	13.00 ±0.4	3.70 ±0.3	1.72 ±0.1	1.21 ±0.1	0.34 ±0.1	0.21 ±0.03	0.27 ±0.04	0.10 ±0.04	0.04 ±0.01	-	-	-	-	-
20	15	75.99 ±0.7	13.50 ±0.4	5.62 ±0.4	1.74 ±0.1	1.80 ±0.1	0.67 ±0.1	0.16 ±0.1	0.26 ±0.04	0.21 ±0.05	0.05 ±0.01	-	-	-	-	-
20	20	73.89 ±1.1	12.31 ±0.7	7.48 ±0.3	1.60 ±0.1	2.35 ±0.2	1.14 ±0.2	0.18 ±0.03	0.38 ±0.1	0.47 ±0.03	0.17 ±0.03	-	0.01 ±0.01	0.01 ±0.01	-	-
20	25	71.68 ±1.0	12.26 ±0.2	9.23 ±0.5	1.47 ±0.04	2.64 ±0.1	1.42 ±0.04	0.16 ±0.1	0.43 ±0.1	0.47 ±0.1	0.23 ±0.1	-	-	-	-	-
20	30	67.10 ±1.8	12.01 ±0.6	11.22 ±0.6	1.60 ±0.2	3.56 ±0.3	1.99 ±0.2	0.23 ±0.02	0.65 ±0.1	0.78 ±0.1	0.38 ±0.1	0.01 ±0.01	0.10 ±0.04	0.16 ±0.1	0.15 ±0.1	0.04 ±0.02
20	50	63.05 ±2.6	9.40 ±0.5	15.21 ±1.1	1.12 ±0.1	4.17 ±0.3	3.82 ±0.4	0.08 ±0.01	0.615 ±0.1	1.45 ±0.1	0.79 ±0.1	-	0.06 ±0.03	0.18 ±0.03	0.22 ±0.02	0.14 ±0.03

Supplementary Table 7. Distribution of Bound Peptides in the Competition Experiment Between Ent1 and Ent2 with Ent2 at a Constant Concentration.

[Ent-1] / μM	[Ent-2] / μM	f 0x / %	f 1x / %		f 2x / %			f 3x / %				f 4x / %				
Ent1/Ent2 peptides bound			1/0	0/1	2/0	"1/1"	0/2	3/0	"2/1"	"1/2"	0/3	4/0	"3/1"	"2/2"	"1/3"	0/4
0	20	87.33 ±0.9	-	11.24 ±0.8	-	-	1.33 ±0.1	-	-	-	0.09 ±0.01	-	-	-	-	-
5	20	81.97 ±0.9	3.25 ±0.1	11.91 ±0.5	0.09 ±0.01	0.80 ±0.1	1.61 ±0.2	-	0.05 ±0.01	0.13 ±0.03	0.18 ±0.04	-	-	-	-	-
10	20	79.71 ±1.0	6.79 ±0.4	9.89 ±1.0	0.48 ±0.1	1.49 ±0.1	1.19 ±0.2	0.01 ±0.01	0.13 ±0.03	0.19 ±0.03	0.13 ±0.01	-	-	-	-	-
15	20	75.70 ±1.1	10.22 ±0.5	9.57 ±0.7	0.87 ±0.1	1.97 ±0.2	1.10 ±0.1	0.05 ±0.01	0.19 ±0.04	0.23 ±0.03	0.10 ±0.01	-	-	-	-	-
20	20	71.80 ±1.2	12.94 ±0.5	8.67 ±0.5	1.66 ±0.1	2.70 ±0.1	1.14 ±0.1	0.17 ±0.03	0.39 ±0.05	0.38 ±0.06	0.15 ± ±0.02	-	-	-	-	-

25	20	71.53 ±2.5	13.97 ±0.9	7.14 ±0.7	2.19 ±0.2	2.70 ±0.3	0.91 ±0.2	0.30 ±0.1	0.58 ±0.1	0.44 ±0.1	0.12 ±0.04	0.02 ±0.01	0.04 ±0.02	0.04 0.03	0.02 ±0.02	0.01 ±0.01
30	20	68.80 ±1.9	16.25 ±1.2	6.97 ±0.3	2.72 ±0.3	2.98 ±0.3	0.89 ±0.1	0.32 ±0.1	0.55 ±0.1	0.36 ±0.03	0.09 ±0.01	0.01 ±0.01	0.03 ±0.03	0.02 ±0.02	0.01 ±0.01	-
50	20	66.39 ±6.8	19.31 ±3.2	5.33 ±0.8	3.97 ±1.2	2.74 ±0.9	0.68 ±0.1	0.525 ±0.3	0.65 ±0.3	0.27 ±0.1	0.05 ±0.02	0.03 ±0.03	0.03 ±0.03	0.04 ±0.04	-	-

Supplementary Methods

Analysis of Native mass spectrometry (native MS) data - Interaction between Clathrin heavy chain (CHC) and the Ent5 peptide.

The processed output of the native MS interaction data consists of the relative fractions of CHC bound to different numbers of peptides. In this case, we detected CHC bound to up to four peptides. From the bound fractions, the total protein concentration, and the total ligand (peptide) concentration, we can calculate the free ligand (peptide) concentration as:

$$[L] = [L_t] - ([CHC \cdot L_1] + 2[CHC \cdot L_2] + 3[CHC \cdot L_3] + 4[CHC \cdot L_4])$$

where $[L_t]$ is the total ligand concentration and $[CHC \cdot L_i]$ is the concentration of CHC bound to *i* ligands.

To average the measured fractions related to similar free ligand concentrations, we used log-spaced intervals with base 1.1 starting at 0.011 µM. Examples of two intervals are [0.011 - 0.0121) µM, centered at 0.0115 µM, and [64.23 - 70.72) µM, centered at 67.4 µM.

The degree of binding (average occupancy) was calculated as:

$$Degree\ of\ binding = \frac{([CHC{\cdot}L_1] + 2[CHC{\cdot}L_2] + 3[CHC{\cdot}L_3] + 4[CHC{\cdot}L_4])}{[P_t]}$$

where [Pt] is the total protein concentration (1.75 µM). The Scatchard plot graphs the degree of binding divided by the free ligand concentration against the degree of binding.

We fitted the five different measured fractions simultaneously to obtain the expected fractions from a four non-interacting binding sites model. The expected concentration of CHC (free protein) is given by

$$[CHC] = [P_t] / (1 + \gamma)$$

where

$$\gamma = \frac{[L]}{k_1} + \frac{[L]^2}{k_1k_2} + \frac{[L]^3}{k_1k_2k_3} + \frac{[L]^4}{k_1k_2k_3k_4}$$

where k_i is the macroscopic dissociation constant of the ith binding event. k_i depends on the microscopic dissociation constant (k) as follows:

$$k_i = k \frac{\Omega_{4,i-1}}{\Omega_{4,i}}$$

where $\Omega_{4,i}$ is the number of microstates for *i* bound ligands and four binding sites. The microscopic dissociation constant (k) is the only fitted parameter.

Finally, the concentration of [CHC · L_i] is calculated as:

$$[CHC \cdot L_i] = [CHC]\,([L]\,/\,k)^i \prod_{j=1}^{i} (4 - j + 1)\,/\,j$$

References

1. Yuen, K. K. The two-sample trimmed t for unequal population variances. *Biometrika* **61**, 165–170 (1974).
