# Supplementary material for: Subtleties in Clathrin heavy chain binding boxes provide selectivity among adaptor proteins of budding yeast
Source: Nat Commun. 2024 Nov 7;15:9655. doi: 10.1038/s41467-024-54037-z (PMC11543927; doi:10.1038/s41467-024-54037-z)
Supplement: Supplementary file 2 — Reporting Summary [file 41467_2024_54037_MOESM2_ESM.pdf]

nature portfolio

Corresponding author(s): Garcia-Alai, Maria

Last updated by author(s): May 30, 2024

Reporting Summary

Nature Portfolio wishes to improve the reproducibility of the work that we publish. This form provides structure for consistency and transparency in reporting. For further information on Nature Portfolio policies, see our Editorial Policies and the Editorial Policy Checklist.

Statistics

For all statistical analyses, confirm that the following items are present in the figure legend, table legend, main text, or Methods section.

n/a	Confirmed	
☐	☒	The exact sample size (*n*) for each experimental group/condition, given as a discrete number and unit of measurement
☐	☒	A statement on whether measurements were taken from distinct samples or whether the same sample was measured repeatedly
☐	☒	The statistical test(s) used AND whether they are one- or two-sided<br>*Only common tests should be described solely by name; describe more complex techniques in the Methods section.*
☒	☐	A description of all covariates tested
☐	☒	A description of any assumptions or corrections, such as tests of normality and adjustment for multiple comparisons
☐	☒	A full description of the statistical parameters including central tendency (e.g. means) or other basic estimates (e.g. regression coefficient) AND variation (e.g. standard deviation) or associated estimates of uncertainty (e.g. confidence intervals)
☐	☒	For null hypothesis testing, the test statistic (e.g. *F*, *t*, *r*) with confidence intervals, effect sizes, degrees of freedom and *P* value noted<br>*Give P values as exact values whenever suitable.*
☒	☐	For Bayesian analysis, information on the choice of priors and Markov chain Monte Carlo settings
☒	☐	For hierarchical and complex designs, identification of the appropriate level for tests and full reporting of outcomes
☒	☐	Estimates of effect sizes (e.g. Cohen's *d*, Pearson's *r*), indicating how they were calculated

Our web collection on statistics for biologists contains articles on many of the points above.

Software and code

Policy information about availability of computer code

Data collection	X-Ray crystallography data was collected in P13 and P14 beamlines operated by EMBL Hamburg in PETRA III, DESY, Hamburg, Germany. MXCube was used for data acquisition.<br>NanoDSF data was collected with PR.ThermControl 2.3.1<br>Fluorescence microscopy data was aquired using NIS Elements 5.42.04

Data analysis	X-ray diffraction data was processed with XDS (0.7.4). Phases were determined using Molecular Replacement (MR) as implemented in MOLREP (11.9.02) with a Phyre2 model of ScCHC-NTD and that particular structure used subsequently for MR of the other complexes. Model building and refinement were done with Coot (0.9.8.1) and REFMAC5 (5.8.0425). In the final steps of model building iSOLDE (1.0b3) was used to improve model geometry quality.<br>For the case of ScCHC-NTD-Apl2.1 complex, PHENIX (1.21_5207) was used for refinement with secondary structure restraints, NCS torsional restraints, TLS refinement and grouped B-factor refinement.<br>NanoDSF Data was analysed using FoldAffinity (spc.embl-hamburg.de)<br>Native Mass Spectrometry data was exported with Xcalibur Qual Browser (Thermo, V4.1.31.9) and all further analysis were done in SigmaPlot (15.0.0.13).<br>Scratchcard plot analysis was used with R with packages tidyverse, minpack.lm and ggpubr.<br>FIJI (v1.54f) and FRETCalc (V 5.0) were used to compute FRET Efficiency.<br>CMEAnalysis (Downloaded from GitHub on 2023.08.28) was used to process and analyze TIRF Microscopy data.<br>Statistical analysis of Fluorescence data was done in R (4.2.3) using packages ggstatsplot for statistical analysis (FRET) and base packages for Mann-Whitney U-test.

For manuscripts utilizing custom algorithms or software that are central to the research but not yet described in published literature, software must be made available to editors and reviewers. We strongly encourage code deposition in a community repository (e.g. GitHub). See the Nature Portfolio guidelines for submitting code & software for further information.

Data

Policy information about availability of data

All manuscripts must include a data availability statement. This statement should provide the following information, where applicable:

- Accession codes, unique identifiers, or web links for publicly available datasets
- A description of any restrictions on data availability
- For clinical datasets or third party data, please ensure that the statement adheres to our policy

All data supporting the findings of this study are available within the paper and its supplementary information. X-ray crystallographic data has been deposited in the PDB with codes 9EXG, 9EX5, 9EXF, 9EXT and 9EYT. The Native mass spectrometry proteomics data have been deposited to the ProteomeXchange Consortium via the PRIDE partner repository with the dataset identifier PXD052864

Research involving human participants, their data, or biological material

Policy information about studies with human participants or human data. See also policy information about sex, gender (identity/presentation), and sexual orientation and race, ethnicity and racism.

Reporting on sex and gender	N/A
Reporting on race, ethnicity, or other socially relevant groupings	N/A
Population characteristics	N/A
Recruitment	N/A
Ethics oversight	N/A

Note that full information on the approval of the study protocol must also be provided in the manuscript.

Field-specific reporting

Please select the one below that is the best fit for your research. If you are not sure, read the appropriate sections before making your selection.

[x] Life sciences [ ] Behavioural & social sciences [ ] Ecological, evolutionary & environmental sciences

For a reference copy of the document with all sections, see nature.com/documents/nr-reporting-summary-flat.pdf

Life sciences study design

All studies must disclose on these points even when the disclosure is negative.

Sample size	For FRET Microscopy: Ent1: Ent1-WT (n=116), Ent1-Cla (n=98), Ent1-Arr (n=102), Ent1-W (n=98), Ent1 Clat+Arr (n=111), Ent1-Clat+W (n=122), Ent1-Arr+W (n=103), Ent1-Cla+Arr+W (n=112). In the case of Ent2, Ent2-WT (n=136), Ent2-Cla (n=98), Ent2-Arr (n=104), Ent2-W (n=126), Ent2-Cla+Arr (n=126), Ent2-Cla+W (n=117), Ent2-Arr+W (n=112), Ent2-Cla+Arr+W (n=127)<br>For TIRF Microscopy: Ent1-WT n=15, Ent1-Cla+Arr n=14 , Ent2-WT n=18, Ent2-Cla+Arr n=13
Data exclusions	No data was excluded for the analysis of the presented study.

Replication	For every Fluorescence microscopy experiment, data was collected on two different days in different microscopy sessions exchanging the order of measurements to account for variability.
Randomization	Not relevant for this study
Blinding	Not blinding was performed, since it was necessary to track which sample corresponded to each type.

Reporting for specific materials, systems and methods

We require information from authors about some types of materials, experimental systems and methods used in many studies. Here, indicate whether each material, system or method listed is relevant to your study. If you are not sure if a list item applies to your research, read the appropriate section before selecting a response.

Materials & experimental systems

n/a	Involved in the study	
☒	☐	Antibodies
☒	☐	Eukaryotic cell lines
☒	☐	Palaeontology and archaeology
☒	☐	Animals and other organisms
☒	☐	Clinical data
☒	☐	Dual use research of concern
☒	☐	Plants

Methods

n/a	Involved in the study	
☒	☐	ChIP-seq
☒	☐	Flow cytometry
☒	☐	MRI-based neuroimaging

Plants

Seed stocks	*Report on the source of all seed stocks or other plant material used. If applicable, state the seed stock centre and catalogue number. If plant specimens were collected from the field, describe the collection location, date and sampling procedures.*
Novel plant genotypes	*Describe the methods by which all novel plant genotypes were produced. This includes those generated by transgenic approaches, gene editing, chemical/radiation-based mutagenesis and hybridization. For transgenic lines, describe the transformation method, the number of independent lines analyzed and the generation upon which experiments were performed. For gene-edited lines, describe the editor used, the endogenous sequence targeted for editing, the targeting guide RNA sequence (if applicable) and how the editor was applied.*
Authentication	*Describe any authentication procedures for each seed stock used or novel genotype generated. Describe any experiments used to assess the effect of a mutation and, where applicable, how potential secondary effects (e.g. second site T-DNA insertions, mosiacism, off-target gene editing) were examined.*
